# Getting Married and Keeping Married

**Published:** 1891-08

**Authors:** 


Getting Married and Keeping Married.

This is number eighteen of the *Human Nature Library*, and the author, who claims to have done both, considers first The Finding of a Mate, in which he considers what should be taken into account in choosing a companion in wedlock and how to do it. There are more than a dozen illustrations, showing Love Signs in mouth, chin, lips, etc., and the suggestions are practical and if followed out would reduce the number of marriage failures. The unmarried should by all means read it, and every married man and woman should read the second part, on Keeping a Mate; the shoals are pointed out on which the marriage bark so often flounders, and the way to keep love fresh and bright is given in a way that must many times prove helpful in promoting happiness that too many know does not always last as it should, in this closest of all relations through life.

It is written in a sprightly and attractive manner, justly placing stress largely on the importance of studying character.

The price of this number is 10 cents. The subscription price of the *Human Nature Library* is 30 cents a year, which may be sent in stamps to Fowler & Wells Co., Publishers, No. 777 Broadway, N. Y.

